# Lister’s Treatment of Wounds by the Antiseptic Method

**Published:** 1876-05

**Authors:** 


### Our Exchanges.

---

Lister's Treatment of Wounds by the Antiseptic Method.—The following lecture, from the *Lancet*, March 25th, 1876, we give somewhat more at length than usual, on account of the great interest of the subject. It is by Thomas Smith, F.R.C.S., Surgeon to St. Bartholomew's Hospital:

The theory, or, one may now say, the facts, on which Mr. Lister's antiseptic treatment rests, are as follows:

1st. That in the dust of the atmosphere, and on matter with which it is in contact, there are the germs of minute organisms which, under favorable circumstances, induce putrefaction in fluids and solids capable of that change, in the same manner as the yeast-plant occasions the alcoholic fermentation in a saccharine solution.

2d. That putrefaction is not occasioned by the chemical action of oxygen or any other gas, but by the fermentative agency of these organisms.

3d. That the vitality or potency of the germs can be destroyed by heat, and by various chemical substances, which we call, in surgery, "antiseptics."

Now, I am not going to ask you to believe these statements on my authority, but I will shortly refer to the results of experiments performed by Pasteur, Lister, Sanderson, Tyndall and others, which justify the above conclusions.

It is scarcely necessary to state that organic fluids, like milk, urine and blood, infusion of meat, etc., if kept in contact with the air at ordinary temperatures, will ere long decompose or putrefy, and will give evidence of putrefaction by turbidity (if the fluid be originally clear) by the evolution of offensive gases, and by the development within them of bacteria.

Further, it has been shown by Pasteur and other observers, that it is by no means essential to the success of such experiments that the organic liquids should be boiled, but that when circumstances admit of their being withdrawn uncontaminated from their natural receptacles, such as the urinary bladder, the blood vessels, the udder of the cow, or the shell of a fresh-laid egg, they will remain free from organisms and from putrefaction when kept in pure vessels and protected from dust.

It has also been discovered that impure air will purify itself by mere subsidence of its dust. Pasteur long ago proved that putrescible fluids could be kept free from putrefaction in air taken from cellars free from draughts, when the solid particles of the atmosphere had had time to deposit themselves by subsidence; and Prof. Tyndall has recently subjected air purified by being kept at rest to very searching tests, to ascertain if it will excite putrefaction in putrescible solutions. He has found that solutions of meat, cheese, turnip, etc., first subjected to high temperature, can be kept free from putrefaction for an indefinite time, exposed to air-closed boxes that have been kept at rest a day or two, to allow the dust to subside, precautions being taken to prevent the said dust rising again by coating the inside of the box with glycerine. The same experimenter has demonstrated the fact that the air which has been thus rendered incapable of exciting putrefaction—i. e., aseptic—is also optically pure; that is, that there are no particles or motes to be detected in it when illuminated by a beam of electric light in a darkened room.

On the other hand, Mr. Lister has found that when any portion of apparatus used in investigations on this subject cannot conveniently be purified by heat, the object may be attained by washing the glass or other material with a strong watery solution of carbolic acid, and drying it with a carbolized rag; and in the course of a long series of experiments he has invariably found this antiseptic agent as efficacious as the flame of a spirit-lamp in preventing the growth of organisms and the occurrence of putrefaction.

Mr Lister's object in the treatment of wounds and abscesses is to exclude from them these germs or organisms that float in the atmosphere and are the causes of putrefaction, and the means he employs for effecting this purpose he recommends, not as the best that can be used, but as the best that he has been able up to the present time to devise; and although Mr. Lister considers the truth of his theory incontrovertible, yet he does not claim to have brought his practice to perfection.

Mr. Lister claims for his plan that when it can be carried out with due care and proper observance of details, he can, as a rule, secure that an open wound should heal after the manner of a subcutaneous injury—that is, without inflammation or constitutional fever, and for the most part without suppuration; while, if suppuration occurs, he secures that it shall not be putrefactive—that is, accompanied by the changes that we consider evidences of putrefaction, such as the formation of bacteria and the evolution of fetid gases.

In the treatment of abscesses by the antiseptic method, Mr. Lister believes that he has effected an entire revolution in the course of the disease after the cavity has been opened, and to this I will more particularly allude in my next lecture. But I may here mention that, along with many local advantages, the patient is said to be free from all danger of irritative fever as the immediate consequence, and from the hectic at a later stage.

I must state these things explicitly to you, in justice to Mr. Lister, that you may judge fairly of the results of the antiseptic treatment, understanding what it cannot do, as well as knowing the advantages claimed for it by its author. It is only just to Mr. Lister, and essential, in order to enable you to form a fair estimate of the results of his method, to remember that he is far from regarding putrefaction as the only cause of suppuration. On the contrary, he has long since pointed out that any antiseptic substance, such as carbolic acid, if applied continuously to the exposed tissues of a wound, stimulates them to granulation, and the granulations to the formation of pus, giving rise to what he has termed "antiseptic suppuration," due to the direct chemical stimulus of the antiseptic. He has also expressed the belief that putrefaction acts in a precisely similar manner in causing suppuration, the products of putrefaction being acrid chemical substances; but there is this all-important difference between the two cases—that the antiseptic acts only on the part to which it is applied, whereas, putrefaction, being a fermentation, extends itself into all the recesses of a wound or abscess, where blood or sloughs, pus or serum, afford a nidus for the development of the bacteria. Further, Mr. Lister has directed attention to the important truth that suppuration, besides being brought about in this manner by the direct stimulus of chemical irritants, may be produced by ordinary inflammation, without the access of any external disturbance, putrefactive or otherwise, as in the familiar case of an ordinary deep-seated abscess, the contents of which, when evacuated, are free from putrefaction. This ordinary inflammation he believes to be due to excited nervous action, and the commonest of all causes of it in surgical practice is tension, occasioned by blood or serum being pent up within the cavity of the wound; and he has insisted upon the fact that, in consequence of the irritating influence of the antiseptic material in the spray and sponges, the sanguineo-serous discharge is greater in the earlier periods from a wound treated antiseptically than from one managed in the ordinary way. Hence it is doubly necessary to provide free escape for this serous effusion, which is done by means of drainage tubes; and if these be neglected or inadequate, tension will inevitably result, with corresponding inflammation, and in due time suppuration. Lastly, we must bear in mind that inflammation caused in this manner by tension, like any other ordinary inflammation, will be attended, in proportion to its intensity, by constitutional disturbance or fever.—*Medical and Surgical Reporter.*

